# Citizenship question effects on household survey response

**DOI:** 10.1002/pam.70004

**Published:** 2025-03-20

**Authors:** J. David Brown, Misty L. Heggeness

**Affiliations:** 1 Senior Researcher for Economic Demography, U.S. Census Bureau, Washington, DC, USA; 2 Associate Professor of Public Affairs and Economics, University of Kansas, Overland Park, Kansas, USA

## Abstract

Differential coverage across demographic groups in a census or survey can reduce the accuracy and representativeness of the resulting statistics. Researchers traditionally have used community-level measures to study response behavior and coverage, which can obscure patterns for small population groups. We illustrate this using household-level citizenship and immigration status. We construct household-level characteristics using administrative records for each address in a randomized control trial (RCT) survey that measured the effects of including a citizenship question on a decennial census questionnaire. Our results show that the self-response rate to the questionnaire without the citizenship question ranges from 70.4% in households with only U.S.-born non-Hispanic Whites to 27.5% in those with at least one likely undocumented person (a 42.9 percentage point gap). Including the citizenship question widens the gap by a statistically significant 2.4 percentage points. Compared to households with all U.S.-born non-Hispanic Whites, the household roster omission rate in households with at least one likely undocumented member is 6.0 times higher without the citizenship question and 10.4 times higher with the question. These patterns help explain why administrative record-based population data include more non-citizens than survey-based official statistics.

## INTRODUCTION

Measuring the accuracy of data on different population groups is important for assessing their representation in statistics, as well as studying the impacts of changes to how the data are collected. Such assessments are difficult to make when survey tests do not collect all characteristics by which researchers may want to assess quality. In addition, no data are collected on nonrespondents, who are key to these assessments. When studying survey nonresponse patterns or making evaluations about characteristics not collected by the survey, researchers typically have linked in characteristics from other sources, aggregated to the community level (Erdman & Bates, 2017; Johnson et al., 2006). Such measures may suffer from a lack of statistical power when used for small population groups such as non-citizens or same-sex couples, groups that researchers suspect are undercounted (O’Hare, 2019).

We illustrate this by studying a household survey designed as a randomized control trial (RCT^1^), called the 2019 Census Test, that measured the effect of including a citizenship question on a decennial census questionnaire.^2^ Sensitivity to the question likely varies by ethnicity, citizenship, immigration status, and national origin, but the 2019 Census Test did not collect data on these characteristics for all sample households. Poehler et al. (2020) produced an official Census Bureau report that studied differences in housing unit response rates with and without the citizenship question. They looked at variation by Hispanic, foreign-born, and non-citizen population shares within the sample household’s tract,^3^ as well as by the historical responsiveness of the tract in decennial censuses.

In this paper, we replicate their results showing that inclusion of the citizenship question does not have a statistically significant effect on the differences in the overall response rate across tract groups as defined by these measures. We then construct household-level characteristics from administrative record (AR) data for each address in the sample and find that the citizenship question lowers response rates for households with at least one likely undocumented member by a statististically significantly larger amount than for households containing only U.S.-born non-Hispanic Whites.

This finding is relevant because even if survey collections include follow-up fieldwork for nonresponse, the fieldwork only partially mitigates biases in the self-response data alone. If a household self-responds to the survey but omits household members, follow-up fieldwork is unlikely to correct for the omission.^4^ We find that in the panel without the citizenship question, household roster omission rates vary from 0.02 people in households containing only U.S.-born non-Hispanic Whites to 0.12 people in ones with at least one likely undocumented member. This difference is statistically significant and helps explain Brown, Heggeness, and Murray-Close’s (2023) finding that AR data contain more non-citizens (especially likely undocumented ones) than leading sources of official statistics on the U.S. population like the American Community Survey (ACS), the 2020 Census, the 2020 Demographic Analysis, and the U.S. Census Bureau’s Population Estimates Program, which serve as benchmarks for many other surveys.

We further find that when adding the citizenship question, the household roster omission rate experiences a statistically significant doubling (0.24 of a person compared to 0.12) in households with at least one likely undocumented member, while the observed change is statistically insignificant in households with all U.S.-born non-Hispanic Whites. Our regression results show that the difference-in-differences estimation (i.e., the difference in the changes) between these two groups is statistically significant. This suggests that including a citizenship question on a decennial census would increase the differential undercount (i.e., the difference in the magnitude of the undercount across groups) of the likely undocumented population relative to U.S.-born non-Hispanic Whites.

The remainder of the paper is structured as follows. The “Literature Review” section discusses findings motivating our research design. The “Data and Methods” section gives a brief overview of how the 2019 Census Test was conducted and how we constructed household characteristics from AR data, describes the methods we employed to analyze the data, and discusses data limitations. We show the results of our analysis for self-response, household roster omissions, and citizenship item nonresponse in the “Results” section. The “Conclusion” section discusses policy implications and gives recommendations for further research.

## LITERATURE REVIEW

Changes to how the decennial census is conducted could affect public policy decisions. O’Hare (2019) has summarized the many ways the decennial census is used in public policy like allocating political power, distribution of federal funds, civil rights enforcement, private business decisions and planning, post-Census population estimates, weights for sample surveys, community planning, economic and social science research, and denominators for various statistics such as the child mortality rate. For example, the inclusion of a citizenship question on the decennial census questionnaire could shift Congressional apportionment. Thomson-Deveaux (2019) has shown the potential effects on the U.S. population distribution across states under different assumptions regarding the undercount of Hispanics, non-citizens, and undocumented immigrants. California, for example, would lose Congressional seats under each of these scenarios.

The representativeness of the decennial census across population subgroups affects the allocation of political power, so making accurate coverage assessments is important. O’Hare (2019) pointed out that decennial census coverage tabulations focus on five demographic characteristics included in the questionnaire (age, sex, race, Hispanic origin, and tenure), neglecting many other characteristics of policy interest, such as housing structure type, immigration status, income, and sexual orientation.

Researchers often study variation in community-level self-response rates by community characteristics for characteristics not included in the decennial census questionnaire.^5^ O’Hare (2015) studied decennial census coverage by characteristics not included in coverage surveys by examining the characteristics of the tracts with the lowest self-response rates to the decennial census, finding that they had disproportionately high shares of people who had less than a high school education, lived below the poverty line, were unemployed, had moved from a different home in the last year, spoke Spanish at home and did not know English very well, were foreign-born, and lived in female-headed households. Erdman and Bates (2017) reported associations between the 2010 Census tract-level self-response rates and shares of the community (measured by block group and tract)^6^ population by different characteristics like age, sex, race, ethnicity, education, income, household size, household composition (e.g., married family), recent in-movers, and housing structure type. Linguistically isolated household, foreign-born, and non-citizen shares of the population did not make the list of the top 25 most influential variables.

Compared to the community-level studies, Brown, Heggeness, and Murray-Close (2023) found clearer indications that non-citizens are underrepresented in the decennial census and other Census Bureau population statistics. Their AR-based data contained 11.0 million (40.8%) more non-citizens than the ACS. When comparing AR to the 2020 Census, they estimated that 19.8% of the AR data’s non-citizens and 30.8% of likely undocumented immigrants were not in the 2020 Census. The AR population is 3.0% higher than the 2020 Census overall, with larger gaps in states with more non-citizens (e.g., 7.6% in California, 5.2% in Nevada, and 5.0% in Texas), which is relevant for Congressional apportionment. The AR Hispanic male population aged 25 to 64, a demographic group containing a high concentration of non-citizens, is 20.1% higher than the 2020 Census. The labor force could thus be larger than the official statistics indicate, which has implications for macroeconomic policy (Edelberg & Watson, 2024). These discrepancies highlight the importance of studying population coverage by citizenship and immigration status in Census Bureau survey tests.

Ethnographic studies provide reasons why non-citizens may be undercounted in the decennial census. De la Puente (1995) found that 1990 Census respondents in households containing non-citizens may have left out people temporarily residing in the household, not in their “nuclear” family, or not related to them. Lee and Ito (2023) reported that some immigrant households in the 2020 Census omitted non-citizen members because they thought the census was only for U.S. citizens. Evans et al. (2019) found that their focus group respondents were likely to omit undocumented immigrants from their 2020 Census responses out of fear that the Census Bureau would share the information with other government agencies.

Commerce Secretary Ross’s March 26, 2018, decision to include a citizenship question on the 2020 Census sparked a flurry of research on the question’s potential effects on 2020 Census data quality. Immediately after the announcement, some observers expressed concern that the question would exacerbate already existing undercounts (e.g., Barry-Jester, 2018). Several new studies have suggested that the inclusion of a citizenship question on the decennial census questionnaire could further reduce non-citizen coverage relative to citizens. Bernhardt and Wunnava (2023) found a 20 to 40 percentage point increase in refusals in the CPS when the survey was redesigned in 1994. One of the changes was the addition of a citizenship question. The authors suggested the citizenship question was a key contributer to the increased refusals, as states with more Hispanics and non-citizens experienced disproportionately large increases precisely in the first month of the redesign. Using the 2018 Census Barriers, Attitudes, and Motivators Study (CBAMS) survey, Walejko et al. (2021) reported that the share of people who said they were “extremely” or “very likely” to respond to the 2020 Census fell by 4.6 percentage points after the March 2018 decision to include a citizenship question on the 2020 Census. The decline was larger by 9.1 percentage points for Hispanics, 8.6 percentage points for foreign-born, 15.8 percentage points for those not speaking English well, and 15.2 percentage points for those responding in Spanish relative to their respective complements.

Some non-citizen groups appear more sensitive to the citizenship question than others. Evans et al. (2019) found that inclusion of a citizenship question would not likely affect the 2020 Census participation of most U.S. citizens, but that it would be detrimental for recent immigrants, especially those without legal status. Latin American–born (LA-born) immigrants would be affected more than other groups, possibly because they were perceived as more likely to be undocumented. Kissam et al.’s (2019) survey suggested that undocumented immigrants would be 55 percentage points less likely to participate in the 2020 Census if it included a citizenship question, a larger drop than for other citizenship and immigration status groups.

Baum et al.’s (2022) citizenship question RCT survey found that item nonresponse rates were negatively affected by the citizenship question among respondents of Mexican or Central American origin, but weak or non-existent among U.S.-born Hispanics or people from Puerto Rico or Cuba. They also showed that among respondents of Mexican or Central American origin, the percentage of the household reported being Hispanic was statistically significantly lower when the citizenship question was included, but the difference was not statistically significant for those from Puerto Rico or Cuba. They suggested that lower reporting of Hispanics could signify Hispanic household roster omissions. Respondents may have reported all members, however, listing some Hispanics as being non-Hispanic. Misclassification would have no bearing on Congressional apportionment, as what matters for that purpose is that people are counted, not how accurately they are classified by demographic groups.

Motivated by the literature above, we extend Poehler et al.’s (2020) 2019 Census Test analysis in several ways. They measured the differences in self-response rates within separate tract-level groups (e.g., tracts with more than 11.1% non-citizens). As we are interested in the citizenship question effects on differential undercount, we measure whether the differences across tract-level groups (e.g., tracts with more than 11.1% non-citizens relative to ones with less than 4.9% non-citizens) are statistically significant. Since tract-level measures could hide underlying patterns in the data, we also conduct analysis using characteristics of the sample households. Several of the studies above suggest that sensitivity to the citizenship question varies among different non-citizen groups, so we separate them into those who had legal status at some point and were born in parts of Latin America, those who had legal status and were born in other countries, and those who were likely undocumented.

Unlike Baum et al. (2022), our household characteristics encompass all household members rather than just the respondent, as they may contain a mixture of citizens and non-citizens with different immigration statuses, each of which may bring a different level of vulnerability.^7^ We study not only housing unit self-response, but also a proxy for household roster omissions, since mixed households might provide data only on their less vulnerable members. We also use the panel without the citizenship question to study how self-response and household roster omission rates vary by household citizenship and immigration status when using the questionnaire later employed in the 2020 Census, which informs the mechanisms behind Brown, Heggeness, and Murray-Close’s (2023) findings of greater AR non-citizen coverage compared to the 2020 Census.

## DATA AND METHODS

The 2019 Census Test data are from a nationally representative random sample of 480,000 housing units. Tracts with high shares of non-citizens and low self-response rates were oversampled. The RCT mirrored the design of the 2020 Census self-response operation. Data collection occurred between June 13 and August 15, 2019, with a reference date of July 1, 2019. The 2020 Census’ Internet First approach, where the first of the five mailings was a postcard with a link to the internet survey instrument, was used in tracts with higher broadband internet penetration. The Internet Choice approach was employed in tracts with lower broadband internet connectivity or with characteristics associated with a low probability of choosing to respond online, and it included a questionnaire and a link to the internet survey instrument in the first mailing.

Besides mail and internet response, Telephone Questionnaire Assistance (TQA) was available, where households could ask questions or receive assistance when encountering technical problems. Interviews were also accepted over the phone. Bilingual English and Spanish materials were sent to households in tracts with high shares of Spanish speakers. The internet instrument was also available in English and Spanish. The questionnaire was the same as in the actual 2020 Census, with the exception that the questionnaires for a randomly selected 240,000 of the housing units also contained the citizenship question that was planned at the time to be added to the 2020 Census. The respondent was asked basic demographic questions about each person in the household, with the citizenship question coming last. Importantly, the citizenship question appeared before the respondent was asked about the second household member. One difference across modes was that people opening the paper questionnaire could immediately see that it either did or did not contain the citizenship question, while those responding online or by phone would not see the question until they reached that point in the instrument. Poehler et al. (2020) have provided further details about the RCT.

The AR come from the Census Bureau’s 2019 Demographic Frame extract.^8^ The data are of 2018 and 2019 vintages. Only records receiving a unique person identifier called a Protected Identification Key (PIK) are included. Only people with Social Security numbers (SSN) or Individual Taxpayer Identification Numbers (ITINs) can receive a PIK.^9^ This means that non-citizens who are ineligible to receive an SSN and who do not file taxes are excluded. The data also include only people with AR addresses that can be linked to the Census Bureau’s Master Address File (MAF) and assigned a Master Address File ID (MAFID). People with more than one MAFID in the 2019 Demographic Frame are assigned the MAFID with the highest person-place probability according to a random forest model predicting the likelihood that a given address is the person’s residence on July 1, 2019.^10^ An important advantage of using AR to construct address characteristics rather than a survey is that they are not influenced by households’ responsiveness to surveys or to the citizenship question, and information is available for both responding and nonresponding addresses.

Table 1 lists all variables used in the analysis. Unlike in past studies on unit self-response, we use variables measured at the housing-unit rather than a higher level of geography like Census tract.^11^ All of the variables are considered hard-to-count or easy-to-count characteristics based on the Erdman and Bates (2017) Low Response Score (LRS) model. The household-level variables related to race, ethnicity, nativity, citizenship, and immigration status are the variables of interest. The controls are “structural” variables capturing living conditions. Kissam and Robinson (2023) argued that measured demographic characteristic associations with responsiveness may reflect the fact that some demographic groups have a greater propensity to live in conditions making them hard to count. We examine this by looking at the extent to which including the controls attenuates the effects of the demographic variables of interest.

Citizenship and place of birth for AR people with SSNs are from the Social Security Administration’s Numerical Identification File (Numident), containing all people issued an SSN. We distinguish LA-born from other foreign-born immigrants, because previous literature has found different response behavior for these groups, as discussed above. LA-born is defined here as being born in Belize, Bolivia, Colombia, Costa Rica, Ecuador, El Salvador, Guatemala, Honduras, Mexico, Nicaragua, or Panama. The race and ethnicity of U.S.-born people in AR are from the Census Best Race File (Ennis et al., 2018). The Census Best Race File consolidates information from AR, household survey data, and past decennial census responses using a set of business rules. ITINs are identified by merging the AR PIKs to the full list of ITIN PIKs. The previous literature discussed above suggests that the households most sensitive to the citizenship question are those with likely undocumented immigrants such as ITIN holders.

Table 2 reports an adjusted Wald test for whether each characteristic’s shares in the panels with and without the citizenship question are statistically significantly different from one another. None of the differences are statistically significant at the 10% level, suggesting that the panels are balanced. Calculations using the numbers in the table indicate that 17.7% of housing units with any AR have at least one non-citizen and 3.7% have at least one ITIN holder. About 79.0% of the households with a non-citizen also have at least one citizen, and 90.2% of those with at least one ITIN holder have at least one person with an SSN.

We study not only unit self-response in the 2019 Census Test, but also the completeness of the response data. One type is whether all household members are included. As mentioned above, Baum et al. (2022) tried to detect household roster omissions by comparing the share of people reported as being Hispanic in their panels with and without a citizenship question, and Poehler et al. (2020) studied the share of the people listed first on the household roster who were reported as Hispanic. Neither study could distinguish between household roster omissions and reclassification of Hispanics as non-Hispanics. We use a more direct measure of household roster omissions, which is the difference between the respondent-reported population count and the number of data-defined people for internet responses with a reported population count of at least 2, and where no people are reported as usually living elsewhere. In such cases, a higher respondent-reported population count indicates that the respondent failed to provide information about some of the people included in their initial population count. We focus on internet responses here, because the respondent does not see all the internet questions until after being asked to provide the population count. In contrast respondents can see all the questions in a paper questionnaire before starting the response. For mail responses we analyze citizenship item nonresponse.


(1) $$y_{i}=\alpha_{i}+\beta_{1}citq_{i}+\beta_{j}\mathrm{Trac}t_{i}+\beta_{k}\mathrm{Trac}t_{i}*citq_{i}+\mu_{i}$$

We estimate logistic and Ordinary Least Squares (OLS) regressions. We first show results from different versions of equation (1), where $y_{i}$ is equal to 1 if household i self-responded to the 2019 Census Test, $citq_{i}$ is an indicator variable equal to 1 if household i received a survey with the citizenship question with the coefficient $\beta_{1}$, $Tract_{i}$ is a vector of explanatory variables in household i’s tract with associated coefficients $\beta_{j}$, $\mathrm{Trac}t_{i}*citq_{i}$ are interaction terms between the tract-level variables and the indicator for household i’s receipt of the citizenship question with coefficients $\beta_{k}$ and $\mu_{i}$ is an error term. We test whether citizenship effects are statistically significantly different across tract groups, a question Poehler et al. (2020) did not investigate. Each regression includes just one type of tract characteristic.

We next turn to household-level variables likely to be associated with sensitivity to the citizenship question, including race, ethnicity, nativity, and immigration status variables. We separately examine associations between each of the household race/ethnicity and citizenship characteristics and response behavior, testing for equality of the coefficients in the samples with and without the citizenship question. This is similar to Poehler et al.’s (2020) analysis testing whether inclusion of the citizenship question affected responsiveness within particular tract groups, but instead we use household-level characteristics.


(2) $$y_{i}=\alpha_{i}+\beta_{1}citq_{i}+\beta_{m}\mathrm{HHsen}s_{i}+\beta_{n}\mathrm{HHsen}s_{i}*citq_{i}+\mu_{i}$$

We then estimate multivariate specifications of equation (2) including all of the household-level characteristics of interest, where $\mathrm{HHsen}s_{i}$ includes household-level indicators for race, ethnicity, citizenship, country of origin, and immigration status, with coefficients $\beta_{m}$, and $\mathrm{HHsen}s_{i}*citq_{i}$ are interaction terms with coefficients $\beta_{n}$. The interactions test whether a group’s difference in responsiveness with compared to without the citizenship question is statistically significantly different than that for all-U.S.-born non-Hispanic White households (the group least likely to be affected by the citizenship question), i.e., a difference-in-differences test. In sum, the multivariate results without interactions show differential responsiveness by household characteristics in the absence of the citizenship question, and the interaction results display how differential responsiveness changes when the citizenship question is present.


(3) $$y_{i}=\alpha_{i}+\beta_{1}citq_{i}+\beta_{m}\mathrm{HHsen}s_{i}+\beta_{n}\mathrm{HHsen}s_{i}*citq_{i}+\beta_{p}HHcont_{i}+\beta_{q}HHcont_{i}*citq_{i}+\mu_{i}$$

Finally, we estimate equation (3), which adds $\mathrm{HHcon}t_{i}$, household-level control variables likely to be associated with self-response, but not likely to be associated with citizenship question sensitivity, together with their associated coefficients $\beta_{p}$. We interact the controls with the citizenship question indicator $\mathrm{HHcon}t_{i}*citq_{i}$ and estimate the associated coefficients $\beta_{q}$.These controls measure the extent to which the associations found in the variables of interest may proxy for “structural” factors (living conditions).

Our study has several limitations. The 2019 Census Test conditions differed from the 2020 Census in important ways. Unlike the 2020 Census, the 2019 Census Test was not accompanied by paid advertising, partnership outreach programs, or significant news coverage, which encourage self-response, especially by internet.^12^ To our knowledge, opponents of the citizenship question did not discourage participation in the RCT, while such an effort may have occurred during the 2020 Census had the question remained on the questionnaire. The decision to include the citizenship question was reversed while the RCT was in the field,^13^ which could have dampened any protest activity. Note that Poehler et al. (2020) did not find any structural breaks in daily response rates in conjunction with these announcements, however. They provided more detailed discussion of the limitations of the 2019 Census Test.

The 2019 Census Test did not include follow-up fieldwork for nonresponding households, unlike in the 2020 Census. Follow-up fieldwork for nonresponding households could mitigate differences in self-response across groups, but as Hill et al. (2022) and O’Hare (2020) showed, substantial differential undercounts remained after fieldwork in the last four decennial censuses.

The AR data may not always place people at their residence on July 1, 2019, either because the AR data do not contain that address, or they also contain other addresses, and one of the others has a higher modelled probability of being the residence. The AR data have incomplete population coverage, e.g., omitting people ineligible for an SSN who did not file taxes with the IRS.^14^ Linkage errors could cause some people to have incorrect demographic characteristics. And others have missing demographic or citizenship/immigration status characteristics. These deficiencies cause inaccuracies in the household characteristics, which may attenuate the measured differences in responsiveness across groups.

## RESULTS

We begin with an analysis of 2019 Census Test unit self-response. The tract-level LRS, percent foreign-born, percent Hispanic, and percent non-citizen variables are strongly negatively associated with unit self-response without the citizenship question (Table 3). Poehler et al. (2020) found statistically significant differences in self-response with and without the citizenship question within tracts with more than 11.1% non-citizens, with high LRS scores, with low LRS scores, and with more than 49.1% Hispanics. Table 3 shows, however, that the change for a category relative to the change in the base category (difference in differences) when the citizenship question is present is insignificant in all cases.

Table 4 shows the 2019 Census Test unit self-response rates separately by household demographic characteristics and by whether the citizenship question is present or not. The response rate to the questionnaire without the citizenship question for households with all-U.S.-born non-Hispanic Whites is 70.4%, compared to 27.5% for households with at least one ITIN holder, a difference of 42.9 percentage points. This is more than twice the 19.3 percentage point gap between tracts with less than 10.6% Hispanics and those with more than 49.1% Hispanics, the 14.3 percentage point gap between tracts with less than 4.9% non-citizens and those with more than 11.1% non-citizens, or the 8.0 percentage point gap between tracts with less than 5.0% foreign-born and those with more than 15.0% foreign-born, as was shown in Poehler et al. (2020).

Turning to other U.S.-born groups, non-Hispanic Blacks and Hispanics have the lowest rates (35.5% and 40.8%, respectively). Households with people born in Latin America have lower rates than other foreign-born, whether they have been naturalized or not. Households with all citizens have higher rates than those with non-citizens with SSNs (64.8% compared to 48.8% for those born outside of Latin America and 34.7% for those born in Latin America).

The top row of Table 4 replicates Poehler et al.’s (2020) result of a small (0.5 percentage point) and statistically insignificant decline in the overall unit self-response rate when the citizenship question is present. Groups with statistically significantly lower response rates with the citizenship question compared to without at the 5% level include households with at least one non-citizen, U.S.-born Hispanic, non-LA-born naturalized citizen, non-citizen with an SSN, an ITIN holder, and households with all non-Hispanic White members. The largest decline (3.18 percentage points) is experienced by households with at least one ITIN holder and at least one SSN holder.

We generally find that the groups with lower self-response rates without the citizenship question have larger drops in rates when the citizenship question is present. The most prominent exception is households with U.S.-born non-Hispanic Blacks, which have one of the lowest rates without the citizenship question (35.5%), but a statistically insignificant difference with the citizenship question (−0.15 percentage points). This is consistent with Evans et al.’s (2019) observation that Blacks in their focus groups had a generally neutral attitude to the question.

The citizenship question effects in Table 4 are much more heterogeneous than those reported by Poehler et al. (2020) using tract characteristics, even when focusing on potentially more sensitive groups. Poehler et al. (2020) found a statistically insignificant 0.6 percentage point lower response rate for households in tracts with more than 15.0% foreign-born people, 0.9 percentage point lower for those with more than 11.1% non-citizens (statistically significant at the 1% level), and 1.1 percentage points lower for those with more than 49.1% Hispanics (statistically significant at the 5% level). Table 4 shows that the response rate drop in the citizenship question panel relative to the panel without the question is 1.26 percentage points for households with non-LA-born naturalized citizens, 1.30 percentage points for those with non-LA-born non-citizens with SSNs, 1.40 percentage points for those with U.S.-born Hispanics, 1.98 percentage points for those with LA-born non-citizens with SSNs, and 3.06 percentage points for those with ITIN holders, all statistically significant at the 5% level.

The share of the sample that is in the group with the largest citizenship question effects (households with at least one ITIN holder) is just 2.4% (Table 2), and only 27.5% of them respond to the questionnaire without the citizenship question. They thus make little contribution to the overall sample of households willing to participate in the RCT (i.e., households who would respond to a questionnaire lacking the citizenship question). That fact and the small effects for other groups help explain why the overall citizenship question effect is insignificant.

In the household-level logistic regressions (Table 5), the differences between each demographic group and all-U.S.-born non-Hispanic White (the base category) without the citizenship question are all statistically significant, except for naturalized citizens, with and without controls. The differences across groups narrow considerably when including controls (e.g., the marginal effect changes from −0.261 to −0.161 for U.S.-born non-Hispanic Blacks), so differences in living conditions captured by the “structural” variables can explain a substantial fraction of the heterogeneity by demographic characteristics. The only statistically significant difference in unit self-response compared to all-U.S-born non-Hispanic Whites when adding the citizenship question is for households with ITIN holders. That effect remains statistically significant at the 1% level with controls, though it declines in absolute value from −0.028 to −0.024. The results suggest that there is differential unit self-response without the citizenship question across these groups, but a differential citizenship question effect is found only in the small group of households most vulnerable to the question. Such narrowly focused effects are not visible when using tract-level variables.

Our proxy for household roster omissions, the difference between the respondent-reported household population count and the number of people for whom sufficient answers are provided, is statistically significantly (1% level) positively associated with all the tract variables. The citizenship question panel has a larger difference in these counts overall and by tract-level characteristics, again statistically significant at the 1% level (Table 6). This suggests the omission rates and the citizenship question effects on them are so heterogeneous across groups that even tract-level variables can identify the patterns.

As shown in Table 7, the average difference between the reported population count and the number of people with answers ranges from 0.02 people for all-U.S.-born non-Hispanic Whites to 0.12 people for households with at least one ITIN holder when the citizenship question is absent. The difference in the full sample increases by a statistically significant (1% level) 0.013 people when the citizenship question is included. The change in the difference when adding the citizenship question is statistically significant for most groups, suggesting broad effects, though it is insignificant for the all-U.S.-born non-Hispanic White household category.

The rates without the citizenship question and the changes when adding the citizenship question are higher in mixed citizen-non-citizen and SSN-ITIN households than in ones with all non-citizens or all ITINs, consistent with Evans et al.’s (2019) reporting that respondents protect more vulnerable household members by omitting them. In multivariate regressions, the only statistically significant (5% level) citizenship question effects relative to all-U.S.-born non-Hispanic White households are for the non-LA-born naturalized citizen, LA-born non-citizen with SSN, and ITIN holder categories (Table 8). This again indicates that the household roster omissions increase when the citizenship question is present is widespread, with particularly large increases for immigrants. The higher rates for LA-born non-citizens with SSNs and ITIN holders (who are often Hispanic) in the citizenship question panel give credence to Baum et al.’s (2022) interpretation that finding fewer Hispanics in the household roster when the question is present is a sign of household roster omissions rather than reclassification of Hispanics as non-Hispanics.

The incidence of complete citizenship item response in mail returns is lower in tracts that are harder-to-count and have more Hispanics, immigrants, and non-citizens (Table 9). The share of households with incomplete citizenship item response is 17.6% for households with a LA-born non-citizen with an SSN, 15.9% for those with an ITIN holder, and 2.8% for all-U.S.-born non-Hispanic Whites (Table 10). These differences are much larger than those for individual citizenship item nonresponse in the ACS, as reported by O’Hare (2018). The differences in rates compared to all-U.S.-born non-Hispanic White households are statistically significant (5% level) for many of the groups without controls, but the effects are noticeably attenuated with controls, and the household with an ITIN holder difference becomes insignificant (Table 11).^15^

## CONCLUSION

We construct AR-sourced household characteristics and combine them with data on responding and nonresponding addresses in the 2019 Census Test. This allows us to analyze differences in responsiveness to a decennial census questionnaire by household type. We also measure differences in responsiveness when adding a citizenship question for each household category. Our results display much wider variation in unit self-response rates across household- than tract-level characteristics. Self-response rates drop by a statistically significantly larger amount in households with at least one ITIN holder than in households with all-U.S.-born non-Hispanic Whites. In contrast, no differential citizenship question effects relative to the base category are found when employing tract-level characteristics like those used by Poehler et al. (2020). This illustrates that using household-level characteristics aids in detecting signs of differential undercounts and changes in them when altering a survey-style collection.

We extend the Poehler et al. (2020) analysis to additional response measures, including household roster omissions and citizenship item nonresponse. There is so much heterogeneity in responsiveness using these measures that we find statistically significant differences even by tract-level characteristics.

Households with ITIN holders are generally least responsive across our measures, LA-born immigrants are less responsive than immigrants from other regions, non-citizens are less responsive than naturalized citizens, and the foreign-born are less responsive than U.S.-born people. The groups that are less responsive to the questionnaire without the citizenship question generally experience larger drops in responsiveness when the citizenship question is included. This suggests that including a citizenship question would exacerbate differential undercounts. Brown, Heggeness, and Murray-Close (2023) found that AR cover more non-citizens, especially ITIN holders and other likely undocumented immigrants, than the 2020 Census and the ACS. The low unit self-response rates and high household roster omission rates reported here for households with non-citizens, particularly those with ITIN holders, illustrate mechanisms through which the lower coverage occurs. This suggests that adding a sensitive question to a survey increases the difficulty of obtaining high-quality data about the subpopulations most sensitive to the question. Data quality may suffer even if large investments are made to ameliorate these challenges.^16^

The linkage of auxiliary data to a survey like what we perform here could fruitfully apply to other survey tests, such as coverage measurement surveys. This would allow researchers to estimate coverage error for a greatly expanded set of characteristics. More generally, researchers could study policy impacts on more precisely identified groups by linking in individual- or household-level auxiliary data to the survey they are using. This approach has the potential to reduce the cost of evaluating statistics and policies.

Our results also provide data-driven information for policymakers interested in receiving the most accurate count of the U.S. population. The addition of sensitive questions like citizenship into a decennial census reduces the total number of people in the count and disproportionately undercounts hard-to-count populations like those without documentation. Methods that harness already existing administrative data can help mitigate that gap.

## Supplementary Material

supplementary appendix

SUPPORTING INFORMATION

Additional supporting information can be found online in the Supporting Information section at the end of this article.

## Figures and Tables

**TABLE 1:** Variable definitions.

**Variable**	**Definition**

**Part A**	**Outcome variables**

2019 Census Test unit self-response	The sample is all addresses in the 2019 Census Test. The value equals 1 if a complete or sufficient partial response is received, and it equals 0 otherwise. The population count could be positive or 0.
Respondent-reported population count minus number of data-defined people	The sample is households where an internet response is chosen, the respondent-reported population count is at least two, the count is at least as large as the number of data-defined people in the response, and no one is reported to usually live elsewhere. The respondent-reported population count is the response to “How many people were living or staying in this house, apartment, or mobile home on July 1, 2019?” This is the first question in the survey. Data-defined people are person-level records in the 2019 Census Test with at least a minimum level of information, indicating an attempt to respond to the survey.
Mail return citizenship item response	The sample is households where a mail return is the chosen response. The dependent variable equals 1 if citizenship question responses are provided for all people in the household roster, and 0 otherwise.

**Part B**	**Explanatory variables of interest**

	**Tract-level explanatory variables of interest**

Low response score	The score predicts the unit self-response rate in the 2010 Census. A higher score is associated with being harder to count, as explained in Erdman and Bates (2017). This is from the 2018 Census Planning Database, containing data from the 2012–2016 American Community Survey.
Tract proportion Hispanic	Hispanic proportion in the tract, from the 2018 Census Planning Database, containing data from the 2012–2016 American Community Survey.
Tract proportion foreign-born	Proportion foreign-born in the tract, from the 2018 Census Planning Database, containing data from the 2012–2016 American Community Survey.
Tract with medium non-citizen share	This is an indicator variable for tracts with between 4.9 and 11.1% non-citizens or a Low Response Score over 24.0, using the 2018 Census Planning Database, containing data from the 2012–2016 American Community Survey.
Tract with high non-citizen share	This is an indicator variable for tracts with more than 11.1% non-citizens using the 2018 Census Planning Database, containing data from the 2012–2016 American Community Survey.

	**Household-level explanatory variables of interest**

All citizen	All the people in AR in the household are U.S. citizens.
Any non-citizen	At least one person in AR in the household is a non-U.S. citizen.
Mixed citizen and non-citizen	At least one person in AR in the household is a U.S. citizen and at least one is a non-U.S. citizen.
All non-citizen	All the people in AR in the household are non-U.S. citizens.
All U.S.-born non-Hispanic White	All the people in AR in the household are U.S.-born non-Hispanic Whites (base category).
Any U.S.-born non-Hispanic Black	At least one person in AR in the household is U.S.-born non-Hispanic Black.
Any U.S.-born non-Hispanic AIAN	At least one person in AR in the household is U.S.-born non-Hispanic American Indian and Alaska Native.
Any U.S.-born non-Hispanic Asian	At least one person in AR in the household is U.S.-born non-Hispanic Asian.
Any U.S.-born non-Hispanic Some Other Race	At least one person in AR in the household is U.S.-born non-Hispanic Some Other Race. We include Native Hawaiian and Pacific Islander in this group.
Any U.S.-born non-Hispanic Two or More Races	At least one person in AR in the household is U.S.-born non-Hispanic Two or More Races.
Any U.S.-born Hispanic	At least one person in AR in the household is U.S.-born Hispanic.
Any LA-born naturalized citizen	At least one person in AR in the household is a naturalized U.S. citizen born in Belize, Bolivia, Colombia, Costa Rica, Ecuador, El Salvador, Guatemala, Honduras, Mexico, Nicaragua, or Panama.
Any non-LA-born naturalized citizen	At least one person in AR in the household is a naturalized U.S. citizen born in a foreign country other than Belize, Bolivia, Colombia, Costa Rica, Ecuador, El Salvador, Guatemala, Honduras, Mexico, Nicaragua, or Panama.
Any LA-born non-citizen with SSN	At least one person in AR in the household is a non-U.S. citizen with an SSN and was born in Belize, Bolivia, Colombia, Costa Rica, Ecuador, El Salvador, Guatemala, Honduras, Mexico, Nicaragua, or Panama and has a Social Security number (SSN).
Any non-LA-born non-citizen with SSN	At least one person in AR in the household is a non-U.S. citizen with an SSN and was born in a foreign country other than Belize, Bolivia, Colombia, Costa Rica, Ecuador, El Salvador, Guatemala, Honduras, Mexico, Nicaragua, or Panama and has a Social Security number (SSN).
Any ITIN	At least one person in AR in the household has an Individual Taxpayer Identification Number (ITIN), which is a 9-digit number in a publicly known range found in the SSN field of AR. They are issued by the Internal Revenue Service (IRS) to people needing to pay taxes, but who are ineligible for an SSN.
Mixed ITIN and SSN	At least one person in AR in the household has an ITIN and at least one has an SSN.
All ITIN	All people in AR in the household have an ITIN.
Any missing race or ethnicity or citizenship	At least one person in AR in the household has missing race, ethnicity, or citizenship.
No administrative data	No people are in AR in the household.

**Part C**	**Control variables**

One person in housing unit	One person is in AR in the household (base category).
Two people in housing unit	Two people are in AR in the household.
Three people in housing unit	Three people are in AR in the household.
Four people in housing unit	Four people are in AR in the household.
Five or more people in housing unit	Five or more people are in AR in the household.
Undeliverable as addressed	The U.S. Postal Service returned one or more of the Census Bureau’s 2019 Census Test mailings to this address with a notification that it was undeliverable as addressed.
Internet first, English questionnaire	The first mailing for this address in the 2019 Census Test was a postcard with a link to reply on the internet. The mailing materials were in English. This was used in tracts with a higher level of internet connectivity and few Spanish speakers (base category).
Internet first, bilingual questionnaire	The first mailing for this address in the 2019 Census Test was a postcard with a link to reply on the internet. The mailing materials were in English and Spanish. This was used in tracts with a higher level of internet connectivity and many Spanish speakers.
Internet choice, English questionnaire	The first mailing for this address in the 2019 Census Test included a paper questionnaire and an invitation to reply on the internet. The mailing materials were in English. This was used in tracts with a lower level of internet connectivity and few Spanish speakers.
Internet choice, bilingual questionnaire	The first mailing for this address in the 2019 Census Test included a paper questionnaire and an invitation to reply on the internet. The mailing materials were in English and Spanish. This was used in tracts with a lower level of internet connectivity and many Spanish speakers.
Married filing jointly	This housing unit had an IRS 1040 tax return of the married filing jointly type.
Unmarried female head of household	This housing unit had an IRS 1040 tax return of the unmarried head of household type, and the filer was female.
Mean number of addresses per person	This is the mean across all AR people in the household of the person’s number of addresses in the 2019 Demographic Frame.
No IRS 1040 return	No Internal Revenue Service (IRS) 1040 return was filed for this household.
Bottom IRS 1040 income quintile	Total monetary income reported in IRS 1040 returns for the household is less than or equal to the 20th percentile of the distribution. This uses the most recent return for each person at the address among returns filed for Tax Year 2017 and 2018. The distribution is calculated using all addresses, whether the address is in the 2019 Census Test or not. Income from 2017 is converted to 2018 dollars using the Bureau of Labor Statistics annual Consumer Price Index for all urban consumers.
Second IRS 1040 income quintile	Total monetary income reported in IRS 1040 returns for the household is above the 20th percentile and less than or equal to the 40th percentile of the distribution.
Third IRS 1040 income quintile	Total monetary income reported in IRS 1040 returns for the household is above the 40th percentile and less than or equal to the 60th percentile of the distribution.
Fourth IRS 1040 income quintile	Total monetary income reported in IRS 1040 returns for the household is above the 60th percentile and less than or equal to the 80th percentile of the distribution.
Top IRS 1040 income quintile	Total monetary income reported in IRS 1040 returns for the household is above the 80th percentile of the distribution.
Single-unit housing structure	The household is in a single-unit housing structure according to the Final Tabulation 2020 Census Master Address File extract (base category).
Multi-unit housing structure	The household is in a multi-unit housing structure according to the Final Tabulation 2020 Census Master Address File extract.
Mobile home or recreational vehicle	The household is in a mobile home or recreational vehicle housing structure according to the Final Tabulation 2020 Census Master Address File extract.
Other unit	The household is in a housing structure other than a single-unit, multi-unit, mobile home, or recreational vehicle. Too few observations are in this category to report separately. This is in the base category together with single-unit structures in the multivariate regressions.
Has person age 0–4	The household contains at least one person age 4 or under in AR.
Has person age 5–17	The household contains at least one person between ages 5 and 17 in AR.
Has person age 18–24	The household contains at least one person between ages 18 and 24 in AR.
Has person age 25–44	The household contains at least one person between ages 25 and 44 in AR (base category).
Has person age 45–64	The household contains at least one person between ages 45 and 64 in AR.
Has person age 65 or over	The household contains at least one person age 65 or over in AR.

**TABLE 2:** Variable percent of initial sample.

	Panel with citizenship question	Panel without citizenship question	Adjusted Wald test Prob > *F*

All citizen	54.70	54.68	0.9215
Any non-citizen	11.77	11.77	0.9665
Mixed citizen and non-citizen	9.27	9.33	0.5123
All non-citizen	2.50	2.44	0.1238
All U.S.-born non-Hispanic White	33.94	33.94	0.9930
Any U.S.-born non-Hispanic Black	10.33	10.13	0.6479
Any U.S.-born non-Hispanic AIAN	0.91	0.92	0.5689
Any U.S.-born non-Hispanic Asian	1.77	1.78	0.7999
Any U.S.-born non-Hispanic Some Other Race	0.41	0.41	0.8074
Any U.S.-born non-Hispanic Two or More Races	2.05	2.01	0.3652
Any U.S.-born Hispanic	11.30	11.35	0.7141
Any LA-born naturalized citizen	1.94	1.96	0.6194
Any non-LA-born naturalized citizen	6.74	6.66	0.3451
Any LA-born non-citizen with SSN	3.41	3.41	0.9543
Any non-LA-born non-citizen with SSN	7.30	7.30	0.9976
Any ITIN	2.44	2.40	0.3982
Mixed ITIN and SSN	2.20	2.17	0.5841
All ITIN	0.24	0.23	0.2579
Any missing race or ethnicity or citizenship	18.17	18.14	0.8192
No administrative data	19.77	19.87	0.5212
One person in housing unit	20.88	20.57	0.0318
Two people in housing unit	22.70	22.83	0.4679
Three people in housing unit	13.04	13.10	0.5947
Four people in housing unit	11.06	11.14	0.3915
Five-plus people in housing unit	12.55	12.50	0.6500
Undeliverable as addressed	9.22	9.27	0.5585
Internet first, English questionnaire	73.54	73.55	0.9406
Internet first, bilingual questionnaire	5.60	5.59	0.9767
Internet choice, English questionnaire	17.24	17.24	0.9987
Internet choice, bilingual questionnaire	3.62	3.62	0.9736
Married filing jointly	36.19	36.43	0.4999
Unmarried female head of household	2.88	2.82	0.3107
Mean number of addresses per person	1.60	1.60	0.5682
No IRS 1040 return	12.25	12.19	0.6440
Bottom IRS 1040 income quintile	13.21	13.22	0.9685
Second IRS 1040 income quintile	13.88	13.94	0.6324
Third IRS 1040 income quintile	14.13	13.98	0.1761
Fourth IRS 1040 income quintile	14.48	14.63	0.2273
Top IRS 1040 income quintile	14.69	14.59	0.4836
Single-unit housing structure	58.00	57.97	0.9316
Multi-unit housing structure	16.01	15.90	0.6364
Mobile home or recreational vehicle	3.57	3.61	0.6116
Has person age 0–4	9.49	9.45	0.6588
Has person age 5–17	21.79	21.66	0.2765
Has person age 18–24	15.41	15.40	0.8955
Has person age 25–44	37.87	37.82	0.8170
Has person age 45–64	39.20	39.37	0.3389
Has person age 65 or over	26.17	26.33	0.4251

*Notes:* The number of observations is 480,000. The adjusted Wald test is based on standard errors from 80 balanced repeated replicate survey weights and a Fay’s adjustment of 0.5. Variable definitions are in Table 1. The data presented in this table are approved for dissemination by the DRB (CBDRB-FY24-CES019–008).

**TABLE 3:** Unit self-response logistic regressions with tract-level variables.

	Marginal effect	Standard error

	Citizenship question panel indicator regression	
Has citizenship question	−0.0046	0.0033
	Tract Low Response Score regression	
Has citizenship question	−0.0061	0.0071
Tract Low Response Score	−1.964***	0.0204
Has citizenship question	0.0056	0.0302
	Tract Hispanic share regression	
Has citizenship question	−0.0033	0.0048
Tract proportion Hispanic	−0.3038***	0.0072
Has citizenship question	−0.0087	0.0129
	Tract foreign-born share regression	
Has citizenship question	−0.0021	0.0064
Tract proportion foreign-born	−0.3225***	0.0148
Has citizenship question	−0.0203	0.0273
	Tract non-citizen share regression	
Has citizenship question	−0.0032	0.0059
Tract with medium non-citizen share	−0.0446***	0.0037
Has citizenship question	−0.0014	0.0065
Tract with high non-citizen share	−0.1422***	0.0040
Has citizenship question	−0.0057	0.0066

*Notes:* Variable definitions are in Table 1. The standard errors use 80 balanced repeated replicate survey weights and a Fay’s adjustment of 0.5. The indented “Has citizenship question” rows are interactions between “Has citizenship question” and the variable immediately preceding it. The number of observations is 480,000. Goodness-of-fit tests are displayed in Appendix Table 2. The data presented in this table are approved for dissemination by the DRB (CBDRB-FY24-CES019-008).

* *p* < 0.10,

** *p* < 0.05,

*** *p* < 0.01.

**TABLE 4:** Percent unit self-response.

	Panel without citizenship question	Panel with citizenship question	Adjusted Wald test Prob > F

All	51.96	51.50	0.1648
All citizen	64.83	64.25	0.1315
Any non-citizen	42.26	40.74	0.0004
Mixed citizen and non-citizen	43.28	41.96	0.0054
All non-citizen	38.37	36.18	0.0122
All U.S.-born non-Hispanic White	70.41	69.74	0.0141
Any U.S.-born non-Hispanic Black	35.46	35.31	0.7352
Any U.S.-born non-Hispanic AIAN	49.77	49.56	0.9089
Any U.S.-born non-Hispanic Asian	62.69	62.93	0.7946
Any U.S.-born non-Hispanic Some Other Race	49.62	51.18	0.5237
Any U.S.-born non-Hispanic Two or More Races	51.19	50.09	0.3442
Any U.S.-born Hispanic	40.83	39.43	0.0038
Any LA-born naturalized citizen	45.54	44.04	0.1312
Any non-LA-born naturalized citizen	57.86	56.60	0.0158
Any LA-born non-citizen with SSN	34.73	32.75	0.0033
Any non-LA-born non-citizen with SSN	48.76	47.46	0.0217
Any ITIN	27.47	24.41	0.0002
Mixed ITIN and SSN	28.63	25.45	0.0002
All ITIN	16.52	15.11	0.5153
Any missing race/ethnicity/citizenship	45.90	45.17	0.1245
No administrative data	24.00	24.35	0.3134

*Notes*: The number of observations is 480,000. The adjusted Wald test for the difference in percentages across panels is based on standard errors from 80 balanced repeated replicate survey weights and a Fay’s adjustment of 0.5. Variable definitions are in Table 1. The data presented in this table are approved for dissemination by the DRB (CBDRB-FY24-CES019-008).

**TABLE 5:** Unit self-response logistic regressions with household-level variables.

	No controls		With controls	
	Marginal effect	Standard error	Marginal effect	Standard error

Has citizenship question	−0.0051**	0.0026	−0.0069	0.0069
Any U.S.-born non-Hispanic Black	−0.2606***	0.0032	−0.1605***	0.0032
Has citizenship question	0.0039	0.0042	0.0055	0.0044
Any U.S.-born non-Hispanic AIAN	−0.1027***	0.0105	−0.0423***	0.0104
Has citizenship question	0.0079	0.0154	−0.0085	0.0139
Any U.S.-born non-Hispanic Asian	0.0395***	0.0074	0.0142**	0.0064
Has citizenship question	0.0145	0.0092	0.0138*	0.0083
Any U.S.-born non-Hispanic Some Other Race	−0.0607***	0.0172	−0.0540***	0.0167
Has citizenship question	0.0271	0.0217	0.0236	0.0210
Any U.S.-born non-Hispanic Two or More Races	−0.0548***	0.0080	−0.0181***	0.0070
Has citizenship question	−0.0008	0.0105	−0.0081	0.0091
Any U.S.-born Hispanic	−0.1477***	0.0032	−0.0822***	0.0029
Has citizenship question	−0.0024	0.0050	−0.0019	0.0045
Any LA-born naturalized citizen	0.0115*	0.0063	−0.0249***	0.0059
Has citizenship question	−0.0043	0.0101	−0.0092	0.0092
Any non-LA-born naturalized citizen	−0.0057	0.0043	−0.0403***	0.0038
Has citizenship question	−0.0067	0.0055	−0.0039	0.0049
Any LA-born non-citizen with SSN	−0.1291**	0.0532	−0.1047***	0.0049
Has citizenship question	−0.0081	0.0079	−0.0056	0.0073
Any non-LA-born non-citizen with SSN	−0.1141***	0.0037	−0.0969***	0.0035
Has citizenship question	−0.0043	0.0062	−0.0047	0.0053
Any ITIN	−0.1903***	0.0062	−0.1385***	0.0054
Has citizenship question	−0.0281***	0.0096	−0.0238***	0.0085
Any missing race/ethnicity/citizenship	−0.0929***	0.0025	−0.0518***	0.0034
Has citizenship question	0.0014	0.0040	−0.0022	0.0056
No administrative data	−0.4253***	0.0029	−0.1962***	0.0052
Has citizenship question	0.0092**	0.0042	0.0104	0.0078

*Notes*: These are marginal effects from logistic regressions with a dependent variable equal 1 if the household responded and 0 otherwise. Variable definitions are in Table 1. The indented “Has citizenship question” rows are interactions between “Has citizenship question” and the variable immediately preceding it. These are marginal effects. The standard errors use 80 balanced repeated replicate survey weights and a Fay’s adjustment of 0.5. The number of observations is 480,000. Goodness-of-fit tests are displayed in Appendix Table 2. The data presented in this table are approved for dissemination by the DRB (CBDRB-FY24-CES019-008).

* *p* < 0.10,

** *p* < 0.05,

*** *p* < 0.01.

**TABLE 6:** Respondent-reported population count minus number of data-defined people OLS regressions with tract-level variables.

	Coefficient	Standard error

	Citizenship question panel indicator regression	
Has citizenship question	0.0126***	0.0022
	Tract low response score regression	
Has citizenship question	−0.0123	0.0087
Tract Low Response Score	0.2128***	0.0273
Has citizenship question	0.1333***	0.0475
	Tract Hispanic share regression	
Has citizenship question	0.0070***	0.0026
Tract proportion Hispanic	0.0792***	0.0101
Has citizenship question	0.0448***	0.0156
	Tract foreign-born share regression	
Has citizenship question	0.0047	0.0030
Tract proportion foreign-born	0.0903***	0.0124
Has citizenship question	0.0675***	0.0194
	Tract non-citizen share regression	
Has citizenship question	0.0089***	0.0027
Tract with medium non-citizen share	0.0154***	0.0039
Has citizenship question	0.0006	0.0052
Tract with high non-citizen share	0.0274***	0.0033
Has citizenship question	0.0240***	0.0063

*Notes:* Variable definitions are in Table 1. The standard errors use 80 balanced repeated replicate survey weights and a Fay’s adjustment of 0.5. The number of observations is 119,000. The data presented in this table are approved for dissemination by the DRB (CBDRB-FY24-CES019-008).

* *p* < 0.10,

** *p* < 0.05,

*** *p* < 0.01.

**TABLE 7:** Mean of respondent-reported population count minus number of data-defined people.

	Panel without citizenship question	Panel with citizenship question	Adjusted Wald test Prob > F

All	0.0332	0.0458	0.0000
All citizen	0.0226	0.0271	0.0374
Any non-citizen	0.0621	0.1083	0.0000
Mixed citizen and non-citizen	0.0671	0.1149	0.0000
All non-citizen	0.0330	0.0691	0.0072
All U.S.-born non-Hispanic White	0.0200	0.0227	0.3301
Any U.S.-born non-Hispanic Black	0.0548	0.0730	0.0837
Any U.S.-born non-Hispanic AIAN	0.0262	0.0257	0.9723
Any U.S.-born non-Hispanic Asian	0.0392	0.0755	0.0026
Any U.S.-born non-Hispanic Some Other Race	0.0370	0.0908	0.1190
Any U.S.-born non-Hispanic Two or More Races	0.0583	0.0646	0.6695
Any U.S.-born Hispanic	0.0714	0.1101	0.0002
Any LA-born naturalized citizen	0.0703	0.1295	0.0113
Any non-LA-born naturalized citizen	0.0352	0.0714	0.0000
Any LA-born non-citizen with SSN	0.0962	0.1944	0.0000
Any non-LA-born non-citizen with SSN	0.0465	0.0773	0.0011
Any ITIN	0.1191	0.2351	0.0001
Mixed ITIN and SSN	0.1198	0.2389	0.0001
All ITIN	0.1047	0.1485	0.7158
Any missing race/ethnicity/citizenship	0.0557	0.0825	0.0001
No administrative data	0.0508	0.0570	0.5638

*Notes:* The adjusted Wald test for the difference across panels is based on standard errors from 80 balanced repeated replicate survey weights and a Fay’s adjustment of 0.5. Variable definitions are in Table 1. The number of observations is 119,000. The data presented in this table are approved for dissemination by the DRB (CBDRB-FY24-CES019-008).

**TABLE 8:** Respondent-reported population count minus number of data-defined people OLS regressions with household-level variables.

	No controls		With controls	
	Coefficient	Standard error	Coefficient	Standard error

Has citizenship question	0.0014	0.0025	0.0147	0.0141
Any U.S.-born non-Hispanic Black	0.0239***	0.0074	0.0139*	0.0078
Has citizenship question	0.0077	0.0105	0.0076	0.0109
Any U.S.-born non-Hispanic AIAN	−0.0070	0.0123	−0.0125	0.0121
Has citizenship question	−0.0138	0.0164	−0.0131	0.0165
Any U.S.-born non-Hispanic Asian	0.0045	0.0079	0.0003	0.0081
Has citizenship question	0.0128	0.0131	0.0129	0.0132
Any U.S.-born non-Hispanic Some Other Race	−0.0026	0.0164	−0.0052	0.0161
Has citizenship question	0.0391	0.0350	0.0382	0.0349
Any U.S.-born non-Hispanic Two or More Races	0.0242**	0.0119	0.0173	0.0121
Has citizenship question	−0.0101	0.0151	−0.0104	0.0154
Any U.S.-born Hispanic	0.0311***	0.0063	0.0212***	0.0062
Has citizenship question	0.0130	0.0097	0.0118	0.0100
Any LA-born naturalized citizen	0.0010	0.0134	−0.0036	0.0132
Has citizenship question	0.0085	0.0240	0.0074	0.0244
Any non-LA-born naturalized citizen	0.0012	0.0046	−0.0011	0.0048
Has citizenship question	0.0238***	0.0082	0.0232***	0.0085
Any LA-born non-citizen with SSN	0.0349**	0.0136	0.0273**	0.0139
Has citizenship question	0.0682***	0.0215	0.0662***	0.0216
Any non-LA-born non-citizen with SSN	0.0126**	0.0064	0.0101*	0.0060
Has citizenship question	0.0111	0.0102	0.0102	0.0100
Any ITIN	0.0591***	0.0153	0.0586***	0.0151
Has citizenship question	0.0702**	0.0280	0.0677**	0.0274
Any missing race/ethnicity/citizenship	0.0239***	0.0040	0.0124**	0.0057
Has citizenship question	0.0123*	0.0064	0.0168	0.0109
No administrative data	0.0334***	0.0083	0.0372***	0.0125
Has citizenship question	0.0049	0.0112	−0.0077	0.0185

*Notes:* These are coefficients from OLS regressions with a dependent variable of the respondent-reported population count minus the number of data-defined people in the response. Variable definitions are in Table 1. The indented “Has citizenship question” rows are interactions between “Has citizenship question” and the variable immediately preceding it. The standard errors use 80 balanced repeated replicate survey weights and a Fay’s adjustment of 0.5. The number of observations is 119,000. The data presented in this table are approved for dissemination by the DRB (CBDRB-FY24-CES019-008).

* *p* < 0.10,

** *p* < 0.05,

*** *p* < 0.01.

**TABLE 9:** Mail return citizenship item response logistic regressions with tract-level variables.

	Marginal effect	Standard error

	Tract Low Response Score regression	
Tract low response score	−0.2344***	0.0184
	Tract Hispanic share regression	
Tract proportion Hispanic	−0.0551***	0.0037
	Tract foreign-born share regression	
Tract proportion foreign-born	−0.0880***	0.0054
	Tract non-citizen share regression	
Tract with medium non-citizen share	−0.0135***	0.0029
Tract with high non-citizen share	−0.0322***	0.0020

*Notes:* The dependent variable equals 1 if citizenship question responses are provided for all people in the household roster, and 0 otherwise. Tract variable definitions are in Table 1. The sample is households where a mail return is the chosen response. The standard errors use 80 balanced repeated replicate survey weights and a Fay’s adjustment of 0.5. The number of observations is 34,500. The data presented in this table are approved for dissemination by the DRB (CBDRB-FY24-CES019-008).

* *p* < 0.10,

** *p* < 0.05,

*** *p* < 0.01.

**TABLE 10:** Percent mail return citizenship question nonresponse.

	Percent nonresponse

All	4.49
All citizen	3.37
Any non-citizen	11.61
Mixed citizen and non-citizen	12.10
All non-citizen	9.33
All U.S.-born non-Hispanic White	2.76
Any U.S.-born non-Hispanic Black	6.36
Any U.S.-born non-Hispanic AIAN	5.04
Any U.S.-born non-Hispanic Asian	7.97
Any U.S.-born non-Hispanic Some Other Race	8.54
Any U.S.-born non-Hispanic Two or More Races	7.44
Any U.S.-born Hispanic	10.34
Any LA-born naturalized citizen	12.74
Any non-LA-born naturalized citizen	6.85
Any LA-born non-citizen with SSN	17.56
Any non-LA-born non-citizen with SSN	8.39
Any ITIN	15.92
Mixed ITIN and SSN	16.10
All ITIN	13.84
Any Missing race/ethnicity/citizenship	10.07
No administrative data	4.25

*Notes:* A household is classified as a nonresponse if there is at least one nonresponse to the citizenship question among people in the household roster and a response if there is a response to the citizenship question for all members of the household roster. The percentages are survey-weighted. Variable definitions are in Table 1. The number of observations is 34,500. The data presented in this table are approved for dissemination by the DRB (CBDRB-FY24-CES019-008).

**TABLE 11:** Mail return citizenship question response logistic regressions with household-level variables.

	No controls		With controls	
	Marginal effect	Standard error	Marginal effect	Standard error

Any U.S.-born non-Hispanic Black	−0.0212***	0.0030	−0.0167***	0.0033
Any U.S.-born non-Hispanic AIAN	−0.0029	0.0106	0.0010	0.0106
Any U.S.-born non-Hispanic Asian	−0.0104	0.0072	−0.0040	0.0071
Any U.S.-born non-Hispanic Some Other Race	−0.0180	0.0133	−0.0155	0.0132
Any U.S.-born non-Hispanic Two or More Races	−0.0176**	0.0075	−0.0115	0.0076
Any U.S.-born Hispanic	−0.0240***	0.0034	−0.0159***	0.0036
Any LA-born naturalized citizen	−0.0103**	0.0052	−0.0055	0.0053
Any non-LA-born naturalized citizen	−0.0143***	0.0046	−0.0103**	0.0049
Any LA-born non-citizen with SSN	−0.0400***	0.0050	−0.0297***	0.0051
Any non-LA-born non-citizen with SSN	−0.0221***	0.0042	−0.0160***	0.0044
Any ITIN	−0.0119**	0.0052	−0.0072	0.0055
Any missing race/ethnicity/citizenship	−0.0325***	0.0029	−0.0128***	0.0037
No administrative data	−0.0156***	0.0040	−0.0156***	0.0058

*Notes:* These are logistic regressions with a dependent variable equal to 1 if citizenship question response are provided for all people in the household roster, and 0 otherwise. Variable definitions are in Table 1. These are marginal effects. Standard errors using 80 balanced repeated replicate survey weights and a Fay’s adjustment of 0.5 are in parenthesis. The number of observations is 34,500. The data presented in this table are approved for dissemination by the DRB (CBDRB-FY24-CES019-008).

* *p* < 0.10,

** *p* < 0.05,

*** *p* < 0.01.

## Data Availability

The code that supports the findings of this study is openly available in Harvard Dataverse at https://dataverse.harvard.edu/dataset.xhtml?persistentId = https://doi.org/10.7910/DVN/MINSHN. Public release of the microdata is prohibited by Titles 13 and 26 U.S.C. Researchers may apply to a Federal Statistical Research Data Center (FSRDC) to do a replication study. Approved researchers may access the code and microdata to conduct a replication study within an FSRDC.

## Footnotes

1 All acronyms are defined in Appendix Table 1. The appendix is available at the end of this article as it appears in JPAM online. Go to the publisher’s website and use the search engine to locate the article at http://onlinelibrary.wiley.com.

2 While a citizenship question was ultimately not included on the 2020 Census questionnaire, the Equal Representation Act (H.R. 7109) passed by the U.S. House of Representatives on May 8, 2024, would add a citizenship question to future decennial censuses. The citizenship question is included in national household surveys like the American Community Survey, the Current Population Survey (CPS), and the Survey of Income and Program Participation (SIPP).

3 Tracts contain about 4,000 people on average (U.S. Census Bureau, 2015).

4 The decennial census includes a coverage follow-up (in 2010) or coverage improvement (in 2020) operation that conducts follow-up telephone interviews with a portion of the households where omissions are suspected. In 2010 there was not sufficient funding to conduct follow-up for all cases identified as needing it (E. K. Poehler, 2010). In the housing unit sample common to the 2010 Census and the 2010 Coverage Measurement Survey that had a different reported population count than the number of people about whom information was reported, just 36% had a coverage follow-up interview (Brown et al., 2016). Thomson-DeVeaux (2019) interviewed Nancy Mathiowetz, an expert on survey research, who expressed skepticism that follow-up efforts could fully mitigate the effect of the citizenship question. She said household roster omissions were more of a problem than item nonresponse, noting that the Census Bureau cannot detect whether family members have been omitted from the roster.

5 This use is supported by Hill et al.’s (2022) findings from the 2020 Census Post-Enumeration Survey that tracts in the bottom two deciles of the 2020 Census self-response distribution (but not other decile groups) had a net undercount, and that tract person omission rates were negatively correlated with self-response rates. In addition, O’Hare (2020) has shown that correlations between self-response rates and net undercounts by demographic characteristics and states are in most cases strong and statistically significant in the 1990, 2000, and 2010 Census.

6 Block group is the next lower level below tract in the Census Bureau’s geographical hierarchy.

7 One might expect mixed households to select less vulnerable members to respond to the questionnaire. If we were to use characteristics only of the respondent, we would fail to identify some households with vulnerable members.

8 Sources include Alaska Permanent Fund Division; Census Household Composition Key; Centers for Medicare and Medicaid Services Medicare Enrollment Database; Centers for Medicare and Medicaid Service Transformed Medicaid Statistical Information System; Federal Housing Administration; Department of Housing and Urban Development Public and Indian Housing Information Center, Tenant Rental Assistance Certification System, and Computerized Homes Underwriting Management System; Internal Revenue Service (IRS) 1040, 1099, and 1099-R; Selective Service System registration data; Social Security Administration (SSA) Master Beneficiary Record; SSA Supplemental Security Record and Special Veterans Benefits; Supplemental Nutrition Assistance Programs; Women, Infants, and Children programs; Temporary Assistance for Needy Families programs; U.S. Postal Service National Change of Address; and Veterans Service Group of Illinois third-party data.

9 Brown, Cohen, et al. (2023) found 4.5 million people in AR in 2020 without either an SSN or ITIN, most of whom are non-citizens. We do not have access to the sources or record linkage for the people in AR without SSNs or ITINs for this project. ITINs are nine-digit numbers in a publicly known range found in the SSN field of administrative records. They are issued by the IRS to non-citizens needing to pay taxes, but who are ineligible for an SSN. Most ITIN holders are thought to be undocumented (Hallman, 2018; United States General Accounting Office, 2004). National Immigration Law Center (2022) provided more details about ITINs.

10 Brown, Cohen, et al. (2023) provided details about this model.

11 Note, however, that though the internet choice and internet first crossed with English only and bilingual materials are housing-unit-level variables, they are the same for each housing unit in the tract in the sample. These are determined based on tract-level measures of internet connectivity and Spanish speaking.

12 Pepe and Shia (2023) reported that the 2020 Census self-response rate for housing units in the 2019 Census Test panel without the citizenship question was 60.0% within the first 64 days (the duration of the test) of 2020 Census self-responses, compared to 51.7% in the test, and these units’ 2020 Census internet response rate was 14 percentage points higher.

13 On June 27, 2019, the Supreme Court ruled in *Department of Commerce v. New York* that Commerce Secretary Ross’s decision to include a citizenship question on the 2020 Census violated the Administrative Procedure Act. On July 2, 2019, Secretary Ross announced that a citizenship question would not be included in the 2020 Census.

14 Undocumented immigrants within that group are likely even more sensitive to a citizenship question than taxpaying ITIN holders.

15 Our working paper (Brown & Heggeness, 2024) shows regressions using an alternative proxy for household roster omissions and internet break-offs in the panels with and without the citizenship question. It examines internet responses in the citizenship question panel to measure the extent to which break-offs occur at the citizenship question or the question is skipped. It also shows how ACS self-response rates vary by household characteristics each year between 2016 and 2019.

16 Lee and Ito (2023) and Robinson et al. (2023) reported that the gap between self-response rates in easier-to-count and hard-to-count tracts widened between the 2010 Census and 2020 Census in California, despite great efforts to encourage participation in hard-to-count tracts.
