# Supplementary material for: Citizenship question effects on household survey response
Source: J Policy Anal Manage. Author manuscript; Available in PMC 2026 Aug 15. (PMC13475791; doi:10.1002/pam.70004)
Supplement: Supplementary file 1 [file NIHMS2129180-supplement-supplementary_appendix.pdf]

APPENDIX

Appendix Table 1. Definitions of acronyms.

Acronym	Definition
ACS	American Community Survey
AIAN	American Indian and Alaska Native
AR	Administrative Records
CBAMS	Census Barriers, Attitudes, and Motivators Study
CPS	Current Population Survey
IRS	Internal Revenue Service
ITIN	Individual Taxpayer Identification Number
LA	Latin America
LRS	Low Response Score
MAF	Master Address File
MAFID	Master Address File Identification number
NHPI	Native Hawaiian or Other Pacific Islander
Numident	Numerical Identification file
OLS	Ordinary Least Squares
PIK	Protected Identification Key
RCT	Randomized Controlled Trial
SIPP	Survey of Income and Program Participation
SSA	Social Security Administration
SSN	Social Security number
TQA	Telephone Questionnaire Assistance

Appendix Table 2. Unit self-response logistic regression goodness-of-fit tests.

	Adjusted *F*-Test		Goodness-of-fit test	
	F-Test value	Prob > *F*	*F*-Test value	Prob > *F*
Table 3 citizenship panel indicator	F(1,79) = 1.97	0.165	F(9,71) = 0.00	1.000
Table 3 tract Low Response Score	F(5,75) = 3,817	0.000	F(9,71) = 14.56	0.000
Table 3 tract Hispanic share	F(3,77) = 2,329	0.000	F(9,71) = 29.11	0.000
Table 3 tract foreign-born share	F(3,77) = 1,166	0.000	F(9,71) = 55.60	0.000
Table 3 tract non-citizen share	F(5,75) = 1,202	0.000	F(9,71) = 0.00	1.000
Table 5 no controls	F(27,53) = 2,109	0.000	F(9,71) = 60.41	0.000
Table 5 with controls	F(73,7) = 604.2	0.000	F(9,71) = 11.50	0.000

Notes: The first adjusted *F*-test is for whether all coefficients in the regression = 0. The second is the *F*-adjusted mean residual goodness-of-fit test (Archer and Lemeshow 2006). The regressions use 80 balanced repeated replicate survey weights and a Fay's adjustment of 0.5. The data presented in this table are approved for dissemination by the DRB (CBDRB-FY25-CES019-004).

REFERENCES

Archer, K. J., & Lemeshow, S. A. (2006). Goodness-of-fit test for a logistic regression model fit using survey sample data. *Stata Journal 6*, 97—105. https://journals.sagepub.com/doi/pdf/10.1177/1536867X0600600106
